# Targeting mTOR-CCL20 Signaling May Improve Response to Docetaxel in Head and Neck Squamous Cell Carcinoma

**DOI:** 10.3390/ijms22063046

**Published:** 2021-03-17

**Authors:** Ming-Huei Chou, Hui-Ching Chuang, Yu-Tsai Lin, Ming-Hsien Tsai, Ying-Hsien Kao, I-Chun Lin, Tai-Lin Huang, Fu-Min Fang, Chih-Yen Chien

**Affiliations:** 1 Graduate Institute of Clinical Medical Sciences, Chang Gung University College of Medicine, Kaohsiung 83301, Taiwan; chou0131@cgmh.org.tw; 2 Center for General Education, Cheng-Shiu University, Kaohsiung 83347, Taiwan; 3 Kaohsiung Chang Gung Head and Neck Oncology Group, Cancer Center, Kaohsiung Chang Gung Memorial Hospital, Kaohsiung 83301, Taiwan; entjulia@cgmh.org.tw (H.-C.C.); xeye@cgmh.org.tw (Y.-T.L.); b9302094@cgmh.org.tw (M.-H.T.); victorhtl@yahoo.com.tw (T.-L.H.); fang2569@cgmh.org.tw (F.-M.F.); 4 Department of Otolaryngology, Kaohsiung Chang Gung Memorial Hospital and Chang Gung University College of Medicine, Kaohsiung 83301, Taiwan; 5 Department of Medical Research, E-Da Hospital, Kaohsiung 82445, Taiwan; ed105156@edah.org.tw; 6 Department of Pediatrics, Kaohsiung Chang Gung Memorial Hospital and Chang Gung University College of Medicine, Kaohsiung 83301, Taiwan; mslinichun@gmail.com; 7 Department of Hematology-Oncology, Kaohsiung Chang Gung Memorial Hospital and Chang Gung University College of Medicine, Kaohsiung 83301, Taiwan; 8 Department of Radiation Oncology, Kaohsiung Chang Gung Memorial Hospital and Chang Gung University College of Medicine, Kaohsiung 83301, Taiwan; 9 Institute for Translation Research in Biomedicine, Kaohsiung Chang Gung Memorial Hospital, Kaohsiung 83301, Taiwan

**Keywords:** head and neck squamous cell carcinoma, mTOR-CCL-20, docetaxel, rapamycin, BEZ235

## Abstract

Patients with advanced head and neck squamous cell carcinoma (HNSCC) usually show a dismal prognosis. It is this worthwhile to develop new, effective therapeutic regimens for these patients, such as molecular targeted therapy, which is promising as an alternative or combination treatment for HNSCC. The mammalian target of rapamycin (mTOR) pathway, which plays an important role in the carcinogenesis of HNSCC, is the most frequently activated, and is thus worthy of further investigation. In this study, two human HNSCC cell lines, FaDu and SAS, were evaluated for cell growth with trypan blue staining and tumor growth using an orthotopic xenograft model. The immunohistochemical expression of mTOR in the subcutaneous xenograft model and the inhibitory effects of docetaxel on the growth and state of activation of the PI3K/mTOR pathway were also evaluated and examined by colony formation and Western blot, respectively. Cell proliferation and migration were measured by water-soluble tetrazolium salt (WST-1) and Oris^TM^ cell migration assay, respectively. Furthermore, the effects of rapamycin and BEZ235, a phosphatidylinositol 3-kinases (PI3K) and mTOR inhibitor in combination with docetaxel or CCL20 were evaluated in the FaDu and SAS cells. The results showed that the expression of mTOR was significantly higher in the SAS and FaDu xenograft models than in the control. Docetaxel treatment significantly suppressed HNSCC cell proliferation and migration in vitro via the PI3K/mTOR/CCL-20 signaling pathway. Additionally, when administered in a dose-dependent fashion, mTOR inhibitors inhibited the growth and migration of the HNSCC cells. This combination was synergistic with docetaxel, resulting in almost complete cell growth and migration arrest. In conclusion, docetaxel significantly inhibited HNSCC cell proliferation and migration in vitro via the PI3K/mTOR/CCL-20 signaling pathway. The synergistic and additive activity of mTOR inhibitors combined with docetaxel shows potential as a new treatment strategy for HNSCC.

## 1. Introduction

Head and neck squamous cell carcinoma (HNSCC) ranks as the world’s 6^th^ most common cancer among male patients, according to the GLOBOCAN 2020 database [1], and the treatment outcomes for advanced-stage tumors remain unsatisfactory. The incidence of head and neck cancer is increasing among male patients in Taiwan due to betel-nut chewing and smoking [2]. Thus, there is an extremely high incidence of oral cancer in Taiwan [3]. The treatment of HNSCC includes upfront surgery or upfront radiation therapy/chemoradiation (CRT). Chemotherapy agents that include platinum-based agents and taxanes have been used widely on patients with advanced HNSCC. Cisplatin is the backbone of the chemotherapy agents used to treat HNSCC, but the taxanes have been clinically adopted only in the setting of induction chemotherapy [4,5] or for palliative purposes after the failure of CRT [6,7].

The phosphoinositide 3-kinase (PI3K) pathway is one of the important pathways involved in the development of HNSCC [8]. However, there are also many genetic mutations in this pathway among patients with HNSCC. For example, an increase of function mutations in the phosphatidylinositol-4,5-bisphosphate 3-kinase catalytic subunit (PIK3C) will increase the expression of protein kinase B (AKT) in tumor cells and promote tumor transformation [9]. The activation of this pathway is related to a poorer prognosis and a variety of malignancies. The mTOR is a downstream molecule that can induce the invasion, migration, proliferation, and angiogenesis of cancer cells [7,10]. Hypoxia-inducible factor-1 (HIF-1) has been associated with resistance to chemoradiation therapy (CRT) and poorer outcomes [11]. The mTOR molecule and HIF-1α are involved in the formation of local tumor progression and distant metastasis. Moreover, an activated mTOR is frequently associated with the activation of HIF-1α and it therefore stimulates angiogenesis [12]. Clinically, it also plays a role in the radioresistance of HNSCC [10]. A high expression of mTOR in HNSCC is found to be related to perineural invasion in the tumor [13] and a poorer prognosis compared to cases with low expression [9,14].

The serine–threonine kinase protein of mTOR forms the catalytic center of two distinct types: mTOR complex 1 (mTORC1), and mTOR complex 2 (mTORC2). These two complexes consist of functionally and structurally different proteins that interact with unique mTOR-related proteins. Rapamycin, an mTORC1/2 kinase inhibitor, and BEZ235, a PI3K/mTOR inhibitor, have been used in several kinds of human malignancy with xenograft models, including head and neck cancer. The mTOR inhibitors which were used as a monotherapy for HNSCC improved the partial tumor response in patients that failed in chemotherapy and/or radiation therapy. However, clinical data has shown that the effects of mTOR inhibitors utilized as single agents in cancer treatment are sometimes dampened by several resistance mechanisms. It is necessary to establish a suitable regimen for mTOR inhibitors that could increase treatment efficacy among these patients [15,16]. The uses of a combination of mTOR inhibitors with other pathway inhibitors are under investigation in different tumor types. Hence, it is worthy to explore novel combination strategies based on mTOR inhibition for HNSCC.

Chemokine (C–C motif) ligand 20, or CCL20, which is also called macrophage inflammatory protein-3 (MIP3A), is an inflammatory chemokine that can interact with the C–C motif chemokine receptor 6 (CCR6) to transmit signaling in cells. Several studies have demonstrated that CCL20 is involved in the metastasis of a variety of malignancies, including pancreatic, hepatic, and colorectal carcinomas [14,17,18,19]. According to an in vitro study, the invasion and migration abilities of colon cancer cell lines and the markers of epithelial-to-mesenchymal transition (EMT), could be increased by treating CCL20 [20]. In addition, CCL20 acts as a chemotactic factor to recruit immune cells [21,22]. Thus, it would be interesting to study its role in tumor development clinically. Therefore, this study aimed to target mTOR–CCL20 signaling to increase the tumor response to treatment with docetaxel combined with an mTOR inhibitor, in HNSCC.

## 2. Results

### 2.1. In Vitro Cell Growth and In Vivo Tumor Growth

**Figure 1:**
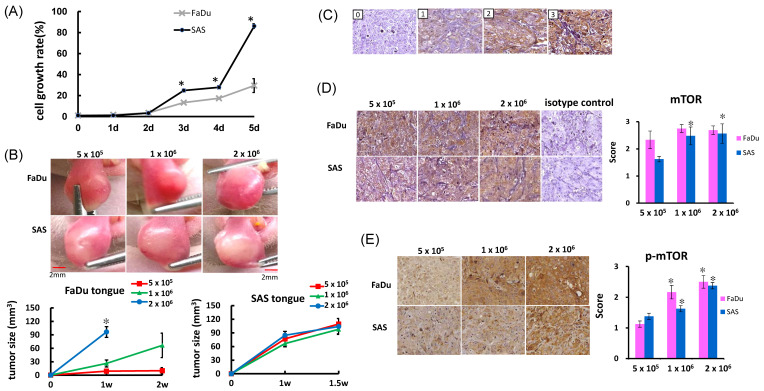
Basal characteristics of FaDu and SAS cells. (**A**) Cell growth was measured by trypan blue staining. Cells (1 × 10^4^ cells in 3 cm culture dishes) were seeded and harvested at different time intervals. After mixing equal volumes of cells and trypan blue, cells were counted by using a hemocytometer. The highest variation in cell growth occurred after 5 days after initiation. Data are expressed as mean ± SD of three separate experiments with triplicate samples. * *p*-value < 0.05 calculated by a paired Student’s t-test. (**B**) NU/NU mice (6 mice per group) received submucosal tongue injections (5 × 10^5^–2 × 10^6^ cells /30μL) of SAS and FaDu cells. The tongue tumors developed 7 days after injection. Tumor size was measured regularly using a digital vernier caliper. (**C**) Expression of the mTOR protein in HNSCC tissues. HNSCC tissues were immunohistochemically stained with an anti-mTOR antibody (×200). 0, no staining; 1, weak staining; 2, intermediate staining; 3, strong staining. (**D**) FaDu and SAS tumor growth was analyzed by mTOR and (**E**) *p*-mTOR immunohistochemistry staining from xenograft tumor tissues (*n* = 6/group) (×200). Mouse mAb IgG1 isotype control, followed by goat anti-mouse IgG1. * *p*-value < 0.05. Error bars: Standard error of the mean (SEM).

In this study, we proposed that docetaxel treatment may induce intracellular and environmental responses resulting in mTOR–CCL20 signaling. First, the growth of HNSCC cells was evaluated by trypan blue staining, and these cells were implanted in the tongues of the tumor growth models [23,24]. In the SAS cells, a significant increase in the cell growth curve was observed on the fifth day. The FaDu cells grew more slowly than the SAS cells (Figure 1A). Tumor volume was measured over a period of 2 weeks post tumor inoculation, or until a volume of 100 mm^3^ was reached. The tumor growth curve (Figure 1B) showed that the tumor volume in the SAS group was significantly larger than in the FaDu group (*p* < 0.01). These results demonstrated that the SAS cells were much larger and more tumorigenic than FaDu cells.

### 2.2. Immunohistochemical Staining for mTOR in NU/NU Mouse Xenograft Models

mTOR signaling mainly regulates cell proliferation and the metabolism involved in tumor initiation and progression [16]. We previously investigated the ways CCL20 increases modulated cell migration and invasion in oral cancer cells. In order to examine the expression of mTOR in the FaDu and SAS xenograft models, the expressions of mTOR and p-mTOR were evaluated by immunohistochemical staining. Irrespective of the number of cells implanted, mTOR expression was significantly higher in the FaDu xenograft models (Figure 1D). However, p-mTOR expression was upregulated with the number of cells implanted in the FaDu xenograft models. Additionally, mTOR and p-mTOR expression were also upregulated with the number of cells implanted in the SAS xenograft models. (Figure 1C,D).

### 2.3. Cytotoxic Activity of Docetaxel on HNSCC Cell Lines

**Figure 2:**
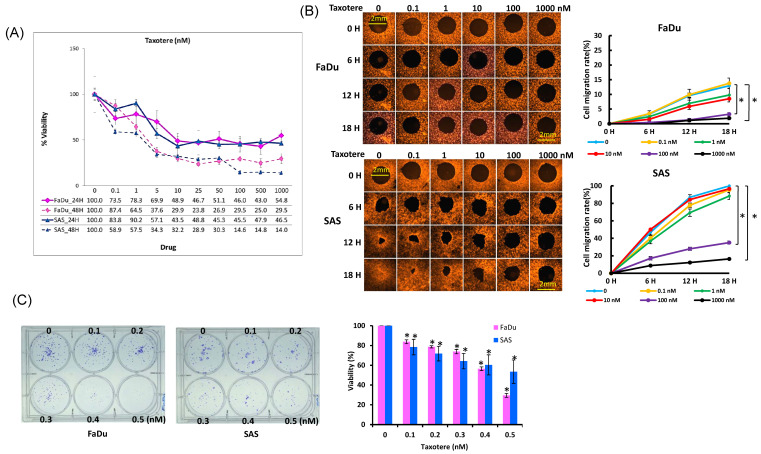
Effects of docetaxel on cell growth and migration. Cells were treated at concentrations ranging from 0.1 to 1000 nM of docetaxel for 24 or 48 h and then treated with WST1 for 3 h. (**A**) FaDu and SAS cell viability was measured by WST-1. Results are the mean ± SD for six independent experiments. (**B**) FaDu and SAS cells were treated at concentrations ranging from 0.1 to 1000 nM of docetaxel for 6 to 18 h, and cell migration was determined by Oris™ Cell Migration Assay. Data are expressed as mean ± SEM (*n* = 6), * *p* < 0.05. (**C**) FaDu and SAS cells were treated at concentrations ranging from 0.1 to 0.5 nM of docetaxel for 10 days, and colony formation was determined by clonogenic assay. Results are the mean ± SD (*n* = 6), * *p* < 0.05 vs. control.

To evaluate the cytotoxic effects of docetaxel, cells were treated with various doses of docetaxel for 24 and 48 h, respectively, and the viability of the cells was measured using a WST1 assay. Docetaxel treatment of the FaDu and SAS cells resulted in a significant decrease in cell viability in a dose-dependent manner during the two-day study period (Figure 2A).

The SAS cells were more sensitive to docetaxel treatment than the FaDu cells. The calculated IC50s (50% inhibitory concentration) were 7.69~2.25 μM and 9.75~3.22 μM for the SAS and FaDu cells, respectively, during the two-day study period. Based on these results, the HNSCC cell lines had different levels of sensitivity to the treatment. Moreover, higher IC50 values were obtained in the docetaxel treatment against the FaDu cells than against the SAS cells. To investigate whether docetaxel inhibits HNSCC cell migration, the effect of docetaxel on cell migration was examined. The ability of cells to migrate into an exposed, circular area in the center of a culture dish during a 72 h period was monitored. Our data clearly showed that treatment with docetaxel caused a significant inhibition of SAS cell migration in a concentration-dependent manner (Figure 2B). Between concentrations of 1 and 1000 nM, docetaxel significantly inhibited circular area closure after 12 h. After eighteen hours, the circular area of the untreated SAS cells was completely closed, and the sheet migration rate of the SAS cells was nine times that of the FaDu cells. The effect of docetaxel was less obvious for the FaDu cells, while no migration inhibition was observed below 10 nM and the circular area of the FaDu cells was not closed after 72 h without treatment (Figure S1). Next, to test the effects of docetaxel on HNSCC colony formation, equal numbers of HNSCC cells (300) were seeded, treated with various concentrations of docetaxel (0, 0.1–0.5 nM), and allowed to grow for 11 days. Different colony numbers of the FaDu and SAS cells after the docetaxel treatment were detected in a dose-dependent manner. Compared to the SAS cells, significantly decreased colony formation was observed in the FaDu cells when treated with 0.5 nM docetaxel (*p* = 0.003) (Figure 2C).

### 2.4. Docetaxel Inhibits mTOR-CCL20 Expression in HNSCC Cells

**Figure 3:**
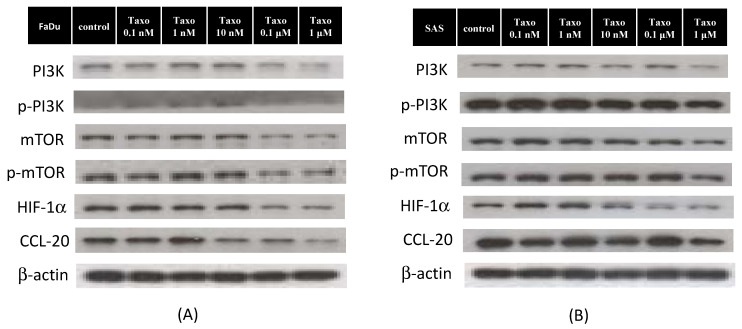
Effect of docetaxel on the mTOR signaling pathway. Cells were treated with docetaxel for 24 h. (**A**) Western blot analysis showed that levels of p-PI3K, PI3K, p-mTOR, mTOR, HIF-1α, and CCL-20 were reduced in FaDu cells treated with docetaxel in a dose-dependent manner. (**B**) In the SAS cell line, the expression of p-PI3K, PI3K, p-mTOR, mTOR, HIF-1α, and CCL-20 were obviously decreased in the group treated with a high dose of docetaxel compared to the control group. Densitometric analysis results are shown on the histograms in Figure S2. Data are expressed as the mean ± SEM of three independent experiments.

Docetaxel is a cytotoxic chemotherapeutic agent that affects HNSCC cell lines, inducing the downregulation of cell proliferation. mTOR is primarily involved in the regulation of cell proliferation and cancer progression. To determine the degree of mTOR activation to which cancer cells exposed to docetaxel were affected by cytotoxicity, the SAS and FaDu cells were incubated with docetaxel for 24 h at different concentrations before being lysed for Western blot analysis. As shown in Figure 3, docetaxel significantly reduced PI3K, mTOR, HIF-1α, and CCL-20 expression at 100 nM in the FaDu cells and at 1000 nM in the SAS cells. The phosphorylation of AKT and extracellular signal-regulated kinase(ERK) was observed to increase significantly in the SAS cells after the 100 nM docetaxel treatment for 24 h, but not in the FaDu cells. Taken together, these data suggest that docetaxel inhibits PI3K/mTOR/CCL-20 signaling, resulting in the inhibition of cell proliferation and migration.

### 2.5. mTOR Inhibitors Enhance the Effects of Docetaxel to Inhibit PI3K/mTOR/CCL-20 Signaling and HNSCC Cell Proliferation/Migration

**Figure 4:**
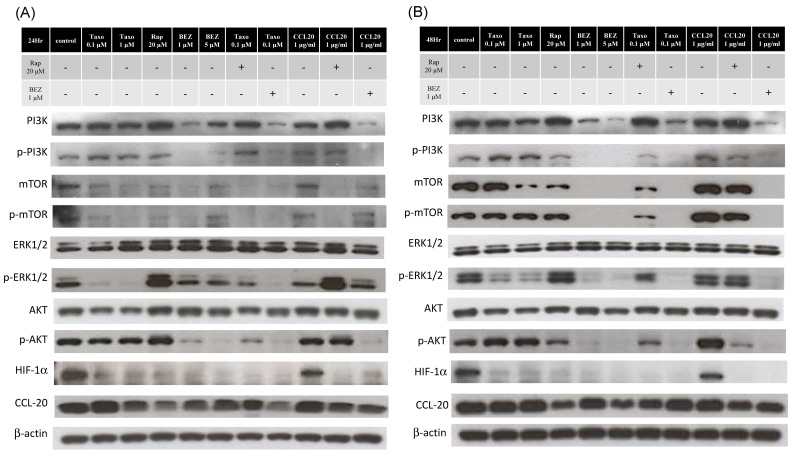

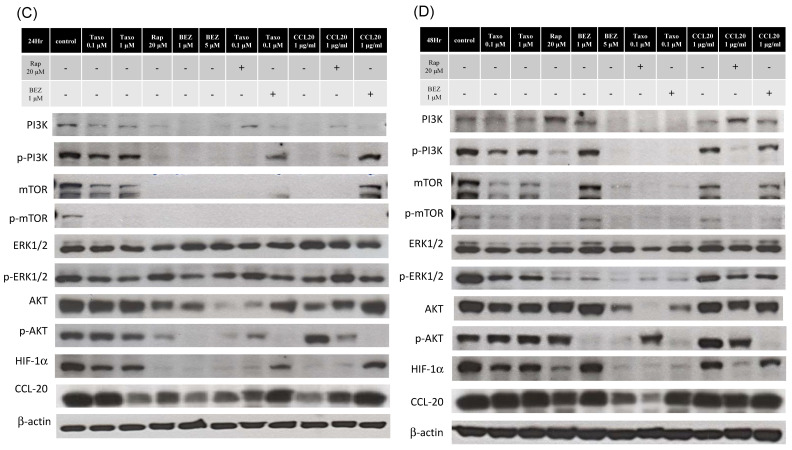
Effect of mTOR inhibitors (rapamycin and BEZ235) combined with docetaxel or CCL-20 on the expression of the mTOR signaling pathway proteins in HNSCC cells. Cells were treated with docetaxel (0.1 or 1 μM), rapamycin (20 μM), BEZ235 (1 or 5 μM), CCL-20 (1 μg/mL), or a combination. After 24 or 48 h, total cell lysates were prepared to perform a Western blot analysis for p-PI3K, PI3K, p-mTOR, mTOR, p-ERK1/2, ERK1/2, p-AKT S473, AKT, HIF-1α, and CCL-20 expression. β-actin was used for loading control. (**A**,**B**) In the FaDu cell line, the marked reduction in mTOR signaling pathway protein levels after treatment with rapamycin or BEZ235 for 24 or 48 h was synergistic with docetaxel and restored by CCL-20 treatment. Unlike BEZ235 (a dual inhibitor of PI3K and mTOR), rapamycin (20 μM) increased p-PI3K and p-ERK1/2 expressions at 24 h. (**C**,**D**) In the SAS cell line, the marked reduction in the p-PI3K, p-ERK1/2, p-AKT S473, and mTOR signaling pathway protein levels after treatment with rapamycin or BEZ235 for 24 or 48 h was synergistic with docetaxel and restored by CCL-20 treatment. The corresponding statistical analysis results for the Western blot tests are shown on the histograms in Figure S3. The data are the mean ± SEM of three independent experiments.

We investigated whether rapamycin and BEZ235 (a novel PI3K–mTOR dual inhibitor) could inhibit either cell growth or the phosphorylation of the downstream targets of the mTOR pathway. In these experiments, the SAS and FaDu cells were treated for 24 h and 48 h with docetaxel, rapamycin, BEZ235, and CCL20 alone, the combination of docetaxel and mTOR inhibitors, or the combination of CCL20 and mTOR inhibitors. As shown in Figure 4, the mTOR inhibitors effectively reduced the levels of mTOR, HIF-1α, and CCL-20 expression in the FaDu and SAS cells. The phosphorylation of PI3K, AKT, and ERK was observed to be significantly reduced in the SAS cells after mTOR inhibitor treatment, and in the FaDu cells after BEZ235 treatment. However, the phosphorylation of AKT and ERK was observed to be significantly increased in the FaDu cells after 20 μM rapamycin treatment for 24 h. The treatment of the FaDu and SAS cells with combined docetaxel and mTOR inhibitors was observed to effectively reduce the expressions of PI3K, mTOR, HIF-1α, CCL-20, as compared to treatment with the docetaxel, CCL20, or mTOR inhibitors alone, or the combination of CCL20 and the mTOR inhibitors.

**Figure 5:**
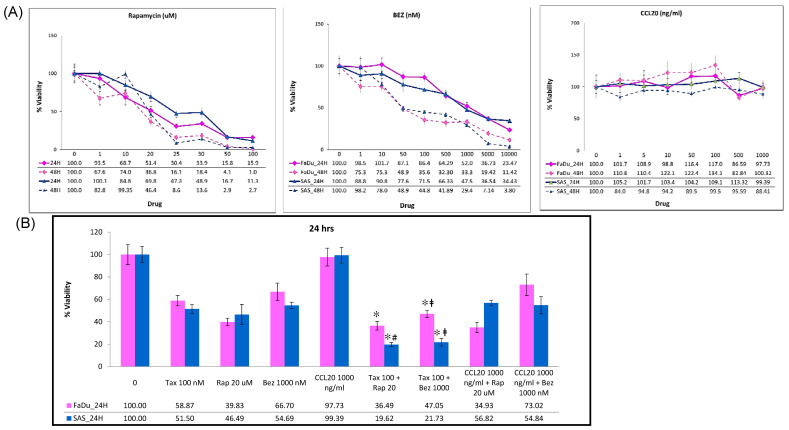
Effect of mTOR inhibitors combined with docetaxel or CCL-20 on cell growth. (**A**) FaDu and SAS cells were treated with rapamycin (1 to 100 μM), BEZ235 (1 to 100 μM), and CCL-20 (1 μg/mL) for 24 or 48 h, and then cells were treated with WST1 for 3 h. Cell viability was determined by measuring absorbance at 450 nm. (**B**) FaDu and SAS cells were treated with docetaxel (0.1 μM), rapamycin (20 μM), BEZ235 (1 μM), CCL-20 (1 μg/mL), or a combination thereof for 24 h, and cell viability was determined by measuring absorbance at 450 nm. Data are expressed as the mean ± SD (*n* = 6). * *p* < 0.05 vs. Tax 100 nM, ^#^ *p* < 0.05 vs. Rap 20 µM, ^‡^ *p* < 0.05 vs. BEZ 1 μM.

To assess the effects of mTOR inhibitors and CCL20 on HNSCC cell growth, we treated the SAS and FaDu cells with different concentrations of rapamycin (1–100 μM), BEZ235 (1–10 μM), or CCL20 (1–1000 ng/mL) using a WST-1 assay. After 24 and 48 h of treatment, the mTOR inhibitors had significant inhibitory and dose-dependent effects on the viability of the SAS and FaDu cells. However, the CCL20 treatment of the SAS and FaDu cells did not produce significant inhibitory or proliferation effects. On the contrary, the FaDu cells were more sensitive to rapamycin at doses in the 30 μM range than the SAS cells. The SAS cells were more sensitive to BEZ235 than the FaDu cells at doses in the 500 nM range (Figure 5A). We examined a combination of chemotherapy agents and mTOR inhibitors to induce synergistic inhibition of the growth of HNSCC cells. The SAS and FaDu cells were treated for 24 h with 100 nM docetaxel, 20 μM rapamycin, 1000 nM BEZ235, 1000 ng/mL CCL20, a combination of docetaxel and mTOR inhibitors, or a combination of CCL20 and mTOR inhibitors. The effects of the combined treatment using docetaxel and the mTOR inhibitors significantly inhibited more cell growth in the FaDu and SAS cell lines than when the cells were treated with docetaxel or mTOR inhibitors alone (Figure 5A,B).

**Figure 6:**
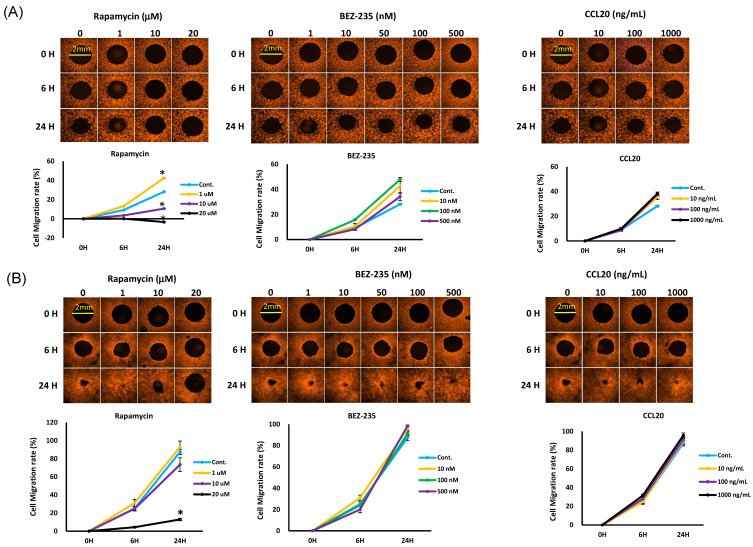

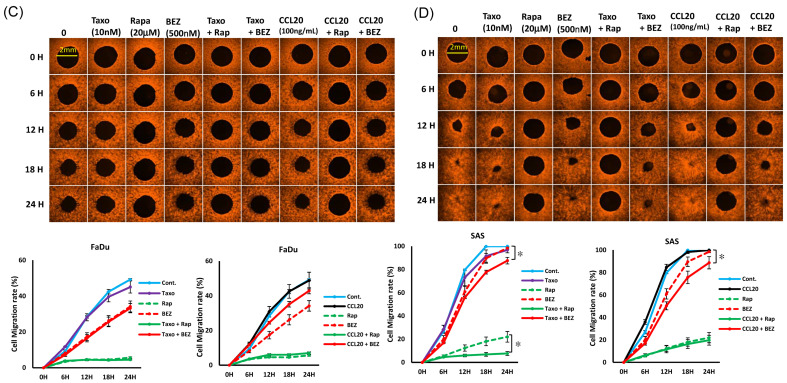
Effect on cell migration of mTOR inhibitors combined with docetaxel or CCL-20. (**A**) FaDu and (**B**) SAS cells were treated with rapamycin (1 to 20 μM), BEZ235 (1 nM to 10 μM), and CCL-20 (0.01 to 1 μg/mL) for 6 to 24 h, and (**C**,**D**) combined with docetaxel or CCL-20 for 6 to 24 h. Cell migration was determined by Oris™ Cell Migration Assay. Data are expressed as the mean ± SEM (*n* = 6). * *p* < 0.05.

In addition, a two-dimensional Oris™ cell migration assay was performed, and the decrease in size of the cell-free area was delayed significantly in the presence of rapamycin or BEZ235, as compared to the solvent-treated SAS and FaDu cells (Figure 6A,B). Rapamycin significantly inhibited cell migration starting at 10 μM. However, the BEZ235 treatment of the SAS and FaDu cells did not produce significant inhibitory effects at doses in the 500 nM range, while CCL20 at higher concentrations (>1000 ng/mL) for 24 h resulted in slight cell proliferation. Due to slower migration, the FaDu cells did not show a different rate of migration under the docetaxel, mTOR inhibitor, or combined treatments. The results also showed that the combined treatment of the SAS cells with docetaxel and the mTOR inhibitors together more effectively inhibited migration than the treatment with docetaxel, CCL20, the mTOR inhibitors alone, or the combination of CCL20 and mTOR inhibitors.

## 3. Discussion

Nowadays, the docetaxel, cisplatin, 5-fluorouracil (TPF) () regimen for induction chemotherapy followed by cisplatin-based concurrent chemoradiation is one of the standard treatments for locally advanced HNSCC [25]. However, many patients still fail with this treatment strategy. The causes of treatment failure involve many factors, such as the defense of the host immune system, the tumor microenvironment, and tumor invasiveness. Taking into account the chemoresistance to taxanes including docetaxel and paclitaxel, patients from an immunohistochemical study who had low expression of class III beta-tubulin isotype in non-small cell lung cancer showed a better response rate, longer progression-free survival, and longer overall survival when treated with paclitaxel [26]. Transducin-like enhancer 3 (TLE3) is a protein related to the tumorigenesis and classification of sarcomas [6,27]. TLE3-positive nuclear staining of breast cancer cells demonstrated a better survival rate when patients underwent systemic combination therapy with taxanes [27]. It remains unclear why some patients with HNSCC respond excellently to chemotherapies, while others fail. The mTOR pathway is a central regulator of mammalian metabolism and physiology that integrates multiple intracellular and extracellular messages, including cell proliferation, angiogenesis, and survival. Thus, mTOR is a key protein in the pathogenesis of tumor growth in multiple organs. The PI3K/AKT/mTOR pathway is upregulated in most HNSCC cells [28], and it is crucial to tumor cell proliferation, differentiation, survival, and metastasis. To date, the adaptation of PI3K/mTOR signaling in response to docetaxel has rarely been described for HNSCC. In the present study, we demonstrated that docetaxel treatment is associated with the downregulation of the PI3K, AKT, mTOR, HIF-α, and CCL20 pathways and the inhibition of proliferation in the SAS and FaDu cells.

mTOR is activated in HNSCC, and it is an attractive therapeutic target clinically [29]. Rapamycin forms a complex with FKBP12 (12 kDa FK506-binding protein), which connects to mTOR and directly inhibits mTORC1 [30]. Rapamycin and rapalogs, including everolimus and temsirolimus, have been used as monotherapies or as a parts of combination therapies for HNSCC in clinical trials [31,32,33] Rapamycin strongly inhibits cell proliferation in numerous murine and human cancer cell lines. It also suppresses hypoxia-mediated angiogenesis and endothelial cell proliferation in vitro [34,35]. In in vivo mouse models, rapamycin displays strong anti-proliferative effects and anti-angiogenic properties, which are associated with decreased vascular endothelial growth factor (VEGF) expression through the inhibition of mTOR signaling [33,36]. The dual PI3K/mTOR inhibitor BEZ235 inhibits PI3K and mTORC1/mTORC2 kinase activity and results in the downregulation of the PI3K/AKT/mTOR pathway [37]. In comparison to the regular PI3K inhibitors, BEZ235 showed strong anti-proliferative and anti-angiogenic activity in breast and renal cancer cell lines.

Here, we showed that the mTORC1 inhibitor rapamycin and a novel PI3K–mTOR dual inhibitor, BEZ235, could inhibit the phosphorylation of downstream targets of the mTOR pathway. TORC1 inhibitors have no direct inhibiting effect on mTORC2 activity and also lead to the feedback activation of the mitogen-activated protein kinase (MAPK) pathways, including AKT and ERK [38,39]. As in our study, rapamycin-induced AKT and ERK feedback activation occurred in the FaDu cells, but not in the SAS cells. By contrast, BEZ235 blocked mTORC1 and mTORC2 activation and inhibited AKT and ERK feedback activation in both the FaDu and the SAS cells. Furthermore, we observed that this pathway could be inhibited in vitro by rapamycin and BEZ235, which resulted in a marked decrease in cell proliferation and reduced cell migration. We also found that the FaDu cells were more sensitive to rapamycin than the SAS cells although rapamycin did induce PI3K, AKT, and ERK feedback activation. On the other hand, the SAS cells were more grown, tumorigenic, and sensitive to BEZ235 than the FaDu cells. Taken together, the findings above suggest that mTOR may present a suitable target for pharmacological intervention in heterogenous HNSCC.

It has been reported that mTOR could positively regulate HIF-1α activation through the PI3K/AKT/mTOR signaling pathway [40]. Moreover, reversible PI3K inhibitors [41] and dual PI3K/mTOR inhibitors [42] could inhibit p-AKT and HIF-1α activation. Our findings suggest that the PI3K/AKT signaling pathway could potentially regulate HIF-1α via mTOR, which could alter the expression of the HIF-1α protein.

Past studies showed that CCL20 was used by cancer cells for physiological functions involving tumorigenesis, angiogenesis, invasion, and metastasis [19,22,43]. Additionally, reports revealed that the AKT, ERK, and MAPK signal pathways could be activated by CCL20, resulting in a significant increase in cell growth and migration. As shown in our results, CCL20 treatment of the SAS and FaDu cells could increase their migration and proliferation effects and restore mTOR signaling pathway activation in HNSCC cells, but the combination of CCL20 and mTOR inhibitor treatment failed to produce these phenomena. It could be that the mTOR signaling pathway regulated the downstream expression of CCL20 in the HNSCC cells.

Furthermore, our results also demonstrated that the treatment of the FaDu and SAS cells with a combination of docetaxel and mTOR inhibitors effectively reduced the expressions of PI3K, mTOR, HIF-1α, CCL-20, and resulted in significant inhibition of cell growth and migration. These results suggest that mTOR inhibitors enhance the anticancer effects of docetaxel in HNSCC.

## 4. Materials and Methods

### 4.1. HNSCC Cell Cultures

The SAS cells were cultured in Dulbecco’s modified Eagle’s medium, F12, containing 4.5 g/mL glucose. (DMEM/F12, Invitrogen, Carlsbad, CA, USA). FaDu cells were cultured in Minimum Essential Medium (MEM medium, Invitrogen, Carlsbad, CA, USA). The culture media were supplemented with 10% fetal bovine serum, 100 μg/mL streptomycin, and 100 U/mL penicillin. Cells (5 × 10^6^ cells in a 10 cm culture dish) were plated and incubated with various concentrations of docetaxel, rapamycin, BEZ235, and CCL-20, or the combination. These were harvested at different time intervals for in vitro study.

### 4.2. Cell Growth Assay

HNSCC cell growth was assessed using trypan blue staining. Cells (seeded at 1 × 10^4^ cells in a 3 cm culture dish) were harvested at different time intervals after treatment of docetaxel, rapamycin, BEZ235, CCL-20, or the combination. After mixing 1:1 with 0.4% trypan blue, cells were counted by using a hemocytometer.

### 4.3. Cell Viability Analysis (WST1)

Cells were plated at 10^4^ cells/well in 96-well plates and cultured with docetaxel, rapamycin, BEZ-235, CCL-20, or the combination for 24–72 h. After treatment, 10 μL of WST-1 (4-[3-(4-Iodophenyl)-2-(4-nitro-phenyl)-2H-5-tetrazolio]-1,3-benzene sulfonate) (Roche Diagnostics, Mannheim, Germany) was added to each well and incubated for an additional 2 h. Absorbance was measured by using a microplate reader at 450 nm and 630 nm.

### 4.4. Clonogenic Assay

The effects of the chemotherapy agents on HNSCC cells to was determined using the colony formation assay. HNSCC cells (300 cells/well) were plated into 6-well plates, incubated with various concentrations of docetaxel, rapamycin, BEZ235, CCL-20, or the combination, and were cultured for 10 days. Colonies were fixed with methanol and stained with crystal violet (0.5% *w*/*v*), and pictures of culture plates were taken using a digital camera. Colonies in each plate were counted and compared with the number of colonies growing in the control cultures. RPE (Relative Plating Efficiency) = [total colonies of three well (test)/total colonies of three well (control)] × 100%.

### 4.5. Western Blot Analysis

Cells were scraped from Petri dishes into PRO-PREP protein extraction solution (iNtRON Bio). Crude protein extracts were collected after centrifugation at 13,000 rpm for 30 min at 4 °C, and the supernatants were quantified using Bradford’s method (Bio-Rad, Hercules, CA, USA). The supernatants containing 20–40 μg of crude proteins were treated with a sample buffer and boiled for 10 min, resolved through 10% SDS-PAGE gels, and transferred to polyvinylidene fluoride membranes. Membranes were blocked with 5% nonfat milk and incubated with primary antibodies targeting PI3K, p-PI3K, AKT, p-AKT, mTOR, p-mTOR, ERK, p-ERK, HIF-1α, CCL20 (1:1000 dilution; Cell Signaling Technology, Danvers, MA, USA), and β-actin (1:10,000 dilution; Abcam, Cambridge, MA, USA) according to the manufacturer’s recommended protocols. Antigenic signals were detected by using a chemiluminescence substrate (Santa Cruz Biotechnology, Dallas, TX, USA), which was exposed to film. Densitometric analysis was performed using Quantity One 1-D software (Bio-Rad, Hercules, CA, USA).

### 4.6. Oris™ Cell Migration Assay

Cell migration assays were measured using an Oris™ cell migration assembly kit (Platypus Technologies, Madison, WI, USA). Cells were plated at a density of 5 × 10^4^ cells/well in Oris™ 96-well plates and were cultured. The Oris™ stoppers were removed after overnight incubation. All wells received CellTracker™ Green and MitoTracker Red to fluorescently stain the cells. Cells were incubated for 72 h to allow for cell migration into the detection zone. The fluorescence signals were measured at various time points using a microplate reader. The graph depicts a real-time cell migration that was acquired from the fluorescence signal present in the detection zones.

### 4.7. Animal Care

The animal experimental procedures were performed and approved by the Institutional Animal Care and Usage Committee of Chang Gung Memorial Hospital in Taiwan (23 October 2014; Approval No:2014092907). All animals were cared for according to the guidelines of the US National Research Council in the Guide for the Care and Use of Laboratory Animals by the Institute of Laboratory Animal Resources.

Male nude (nu/nu) mice, 6 weeks of age, were procured from the National Laboratory Animal Center (Academia Sinica, Taipei, Taiwan) and these mice were housed in a specific pathogen-free (SPF) facility. The animals had free access to experimental mouse diets and autoclaved reverse-osmosis treated water. All of the mice were anesthetized with Zoletil (15 mg/kg body weight).

(1) Orthotopic xenograft model: Male nude mice (6 mice per group) received a submucosal tongue injection [24,25] (5 × 10^5^–2 × 10^6^ cells /30 μL) of SAS and FaDu cells.

The tongue tumors developed 7 days after injection. The two longest perpendicular axes of each tumor surface were routinely monitored by a digital vernier caliper. The mice were measured twice a week for weight loss (Figure S4) and euthanized humanely by CO_2_ inhalation when they had lost more than 25% of their pre-injection body weight.

(2) Necropsy and Tissue Preparation

After the mice were euthanized humanely, the tumors were collected, weighed, and fixed in formalin solution overnight. The specimens were completely embedded in paraffin and 2 μm slices were prepared. Slices were stained with hematoxylin–eosin (H&E) and evaluated by light microscopy.

### 4.8. Statistical Analysis

Data were expressed as the mean ± SEM of at least three experiments for the animal, densitometric, and cell migration data analyses, and as the mean ± SD of at least three in vitro experiments. We used the Student’s *t*-test (unpaired, 2-tailed) to compare the experimental groups with continuous variables and used the Student’s *t*-test and ANOVA to assess differences in the animal groups. Differences were considered significant at *p* < 0.05.

## Data Availability

The data presented in this study are available on request from the corresponding author. The data are not publicly available due to privacy.

## Supplementary Materials

The following are available online at https://www.mdpi.com/1422-0067/22/6/3046/s1.

## Abbreviations

HNSCC	head and neck squamous cell carcinoma
GLOBOCAN	Global Cancer Observatory CANCER TODAY
CRT	chemoradiation therapy
PI3K/AKT/mTOR	phosphoinositide 3-kinase/protein kinase B/mammalian target of rapamycin
CCL20	Chemokine (C-C motif) ligand 20
HIF-1α	Hypoxia-Inducible Factor-1α
