# Supplementary material for: Targeting mTOR-CCL20 Signaling May Improve Response to Docetaxel in Head and Neck Squamous Cell Carcinoma
Source: Int J Mol Sci. 2021 Mar 17;22(6):3046. doi: 10.3390/ijms22063046 (PMC8002492; doi:10.3390/ijms22063046)
Supplement: Supplementary file 1 [file ijms-22-03046-s001.pdf]

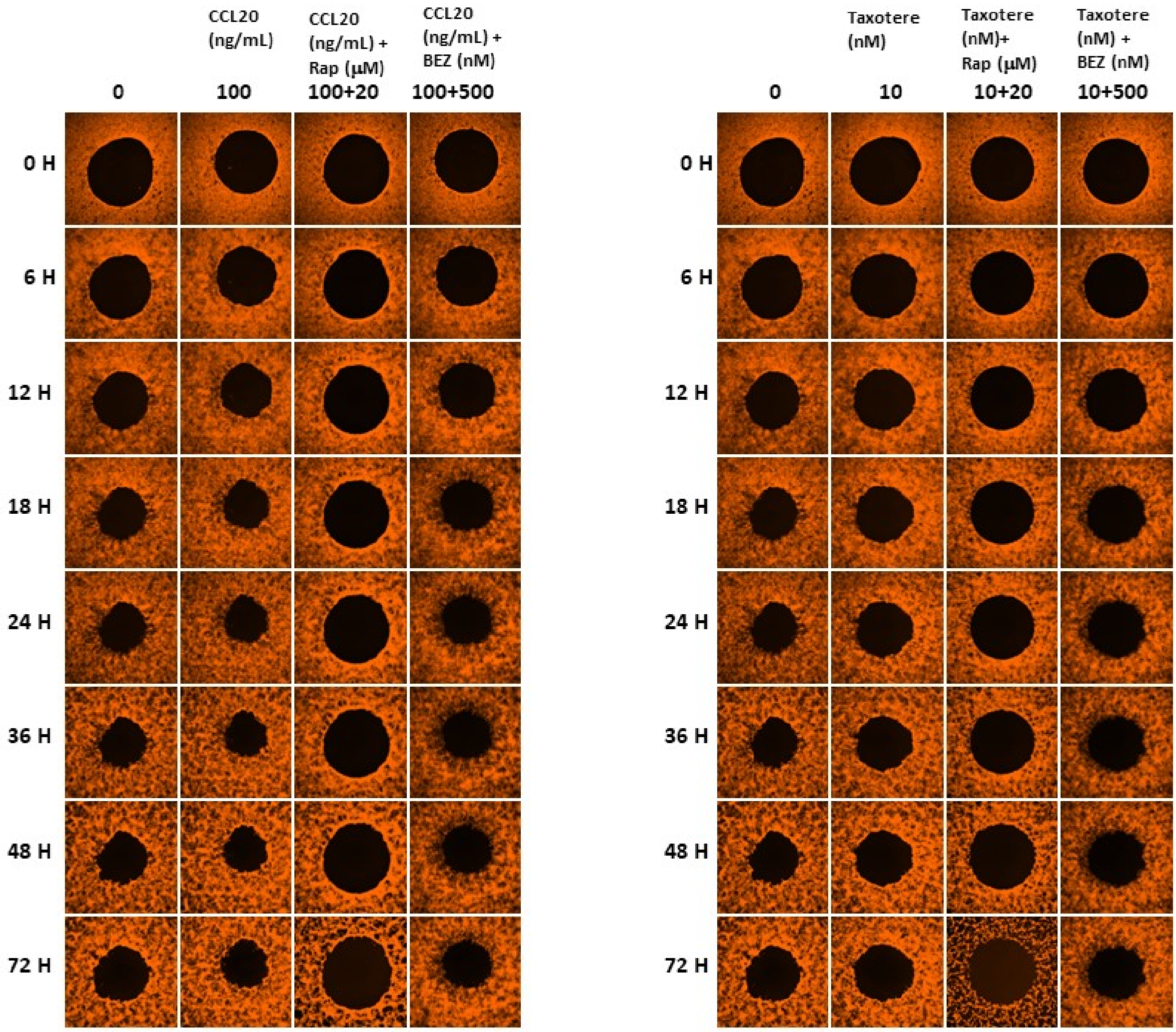

Figure S1. FaDu cells were treated with CCL-20 or docetaxel for for 6 hrs to 72 hrs and combined with rapamycin or BEZ235 for 6 hrs to 72 hrs. Cell migration was determined by Oris™ Cell Migration Assay. The circular area of FaDu cells was not closed after 72 hours with or without treatment.

(A) FaDu

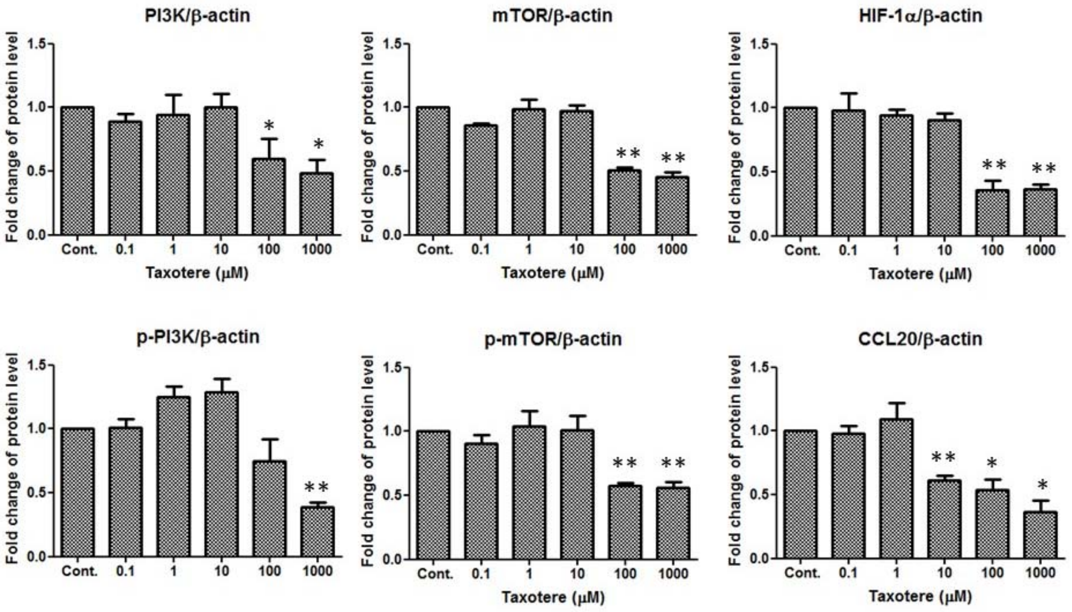

(B) SAS

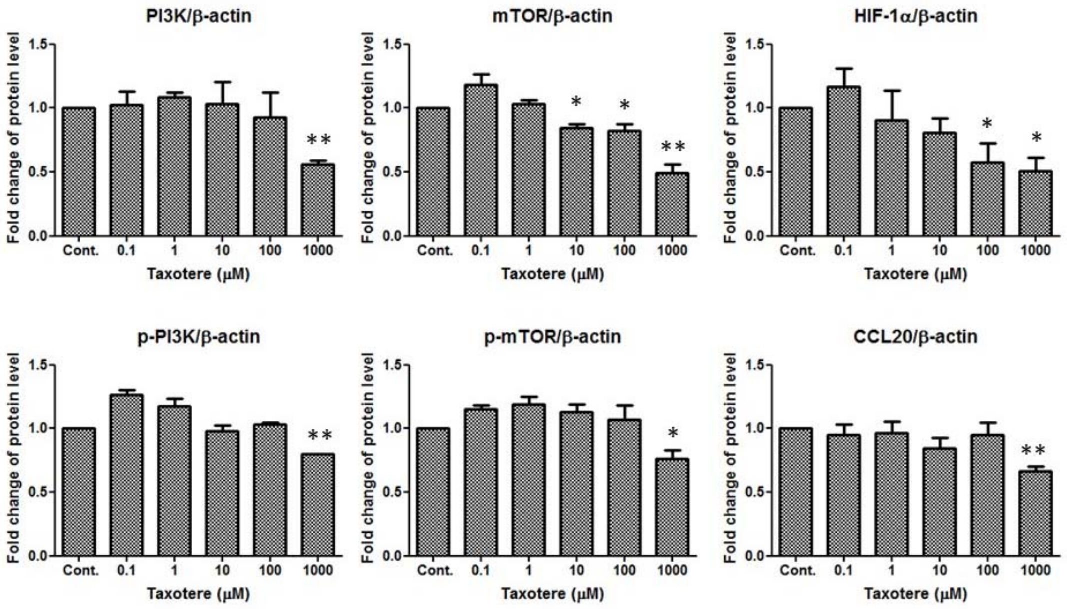

Figure S2. Effect of docetaxel on the mTOR signaling pathway. Cells were treated with docetaxel for 24hrs. (A) Western blot analysis showed that level of p-PI3K, PI3K, p-mTOR, mTOR, HIF-1α and CCL-20 were reduced in FaDu cells treated with docetaxel in a dose-dependent manner. (B) In the SAS cell line, the expression of p-PI3K, PI3K, p-mTOR, mTOR, HIF-1α and CCL-20 were obviously decreased in a group treated with a high dose of docetaxel compared to the control group. Densitometric analysis results are means ± SEM for three independent experiments.

*$p<0.05$, **$p<0.001$

(A) FaDu 24H

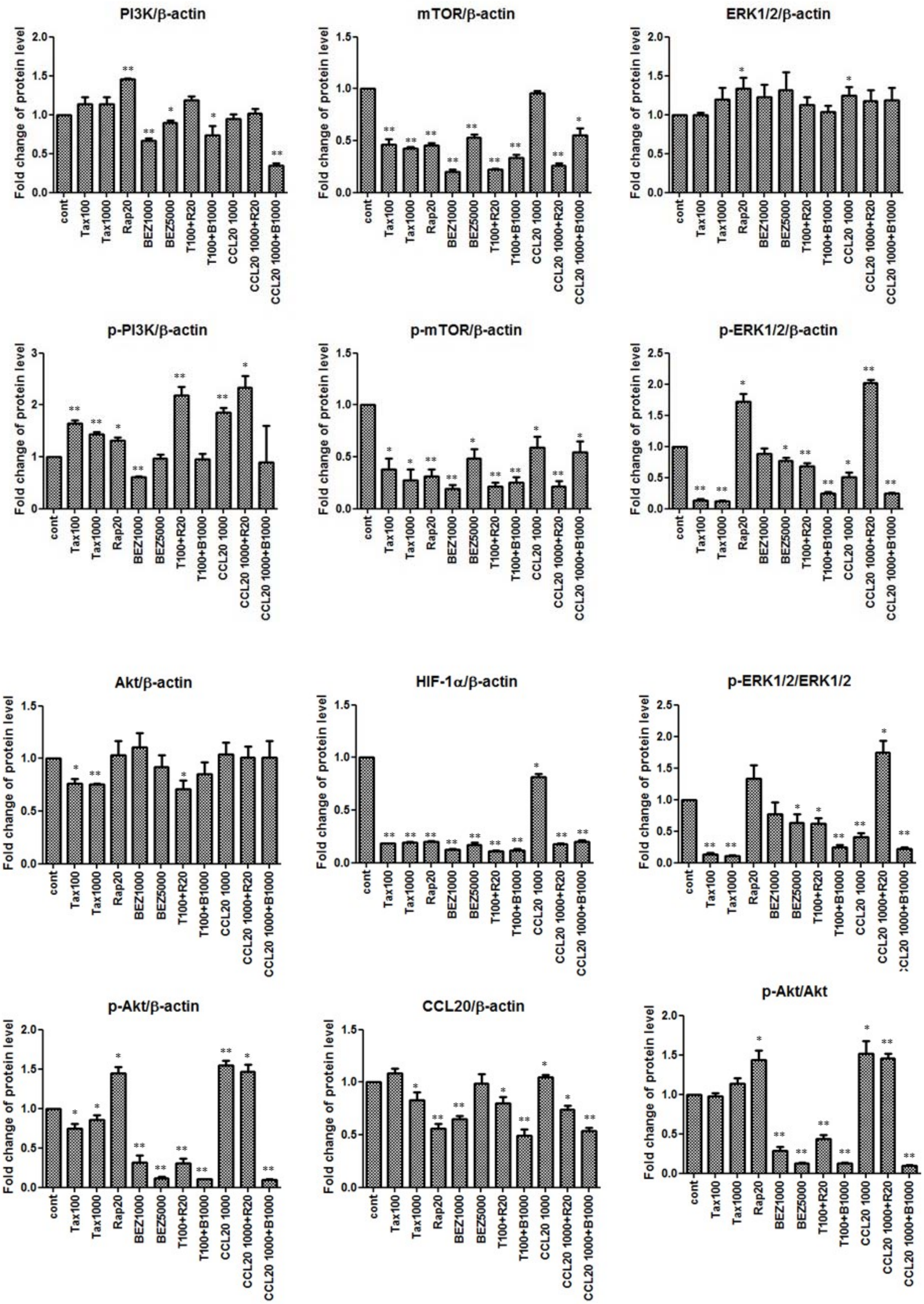
PI3K/β-actin
mTOR/β-actin
ERK1/2/β-actin
p-PI3K/β-actin
p-mTOR/β-actin
p-ERK1/2/β-actin
Akt/β-actin
HIF-1α/β-actin
p-ERK1/2/ERK1/2
p-Akt/β-actin
CCL20/β-actin
p-Akt/Akt
Fold change of protein level
cont
Tax100
Tax1000
Rap20
BEZ1000
BEZ5000
T100+R20
T100+B1000
CCL20 1000
CCL20 1000+R20
CCL20 1000+B1000

(B) FaDu 48H

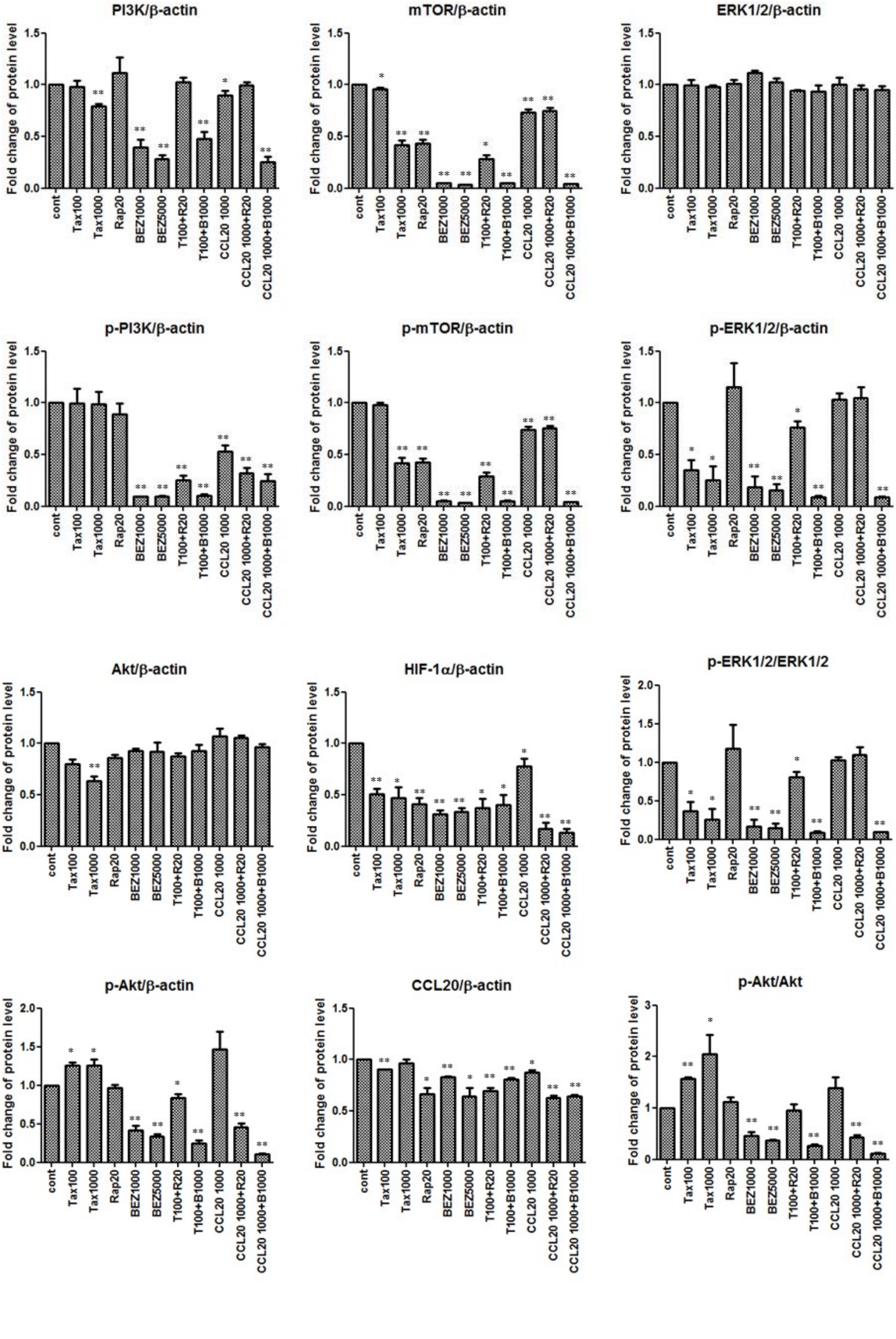

PI3K/β-actin
mTOR/β-actin
ERK1/2/β-actin
p-PI3K/β-actin
p-mTOR/β-actin
p-ERK1/2/β-actin
Akt/β-actin
HIF-1α/β-actin
p-ERK1/2/ERK1/2
p-Akt/β-actin
CCL20/β-actin
p-Akt/Akt
Fold change of protein level
cont
Tax100
Tax1000
Rap20
BEZ1000
BEZ5000
T100+R20
T100+B1000
CCL20 1000
CCL20 1000+R20
CCL20 1000+B1000

(C) SAS 24H

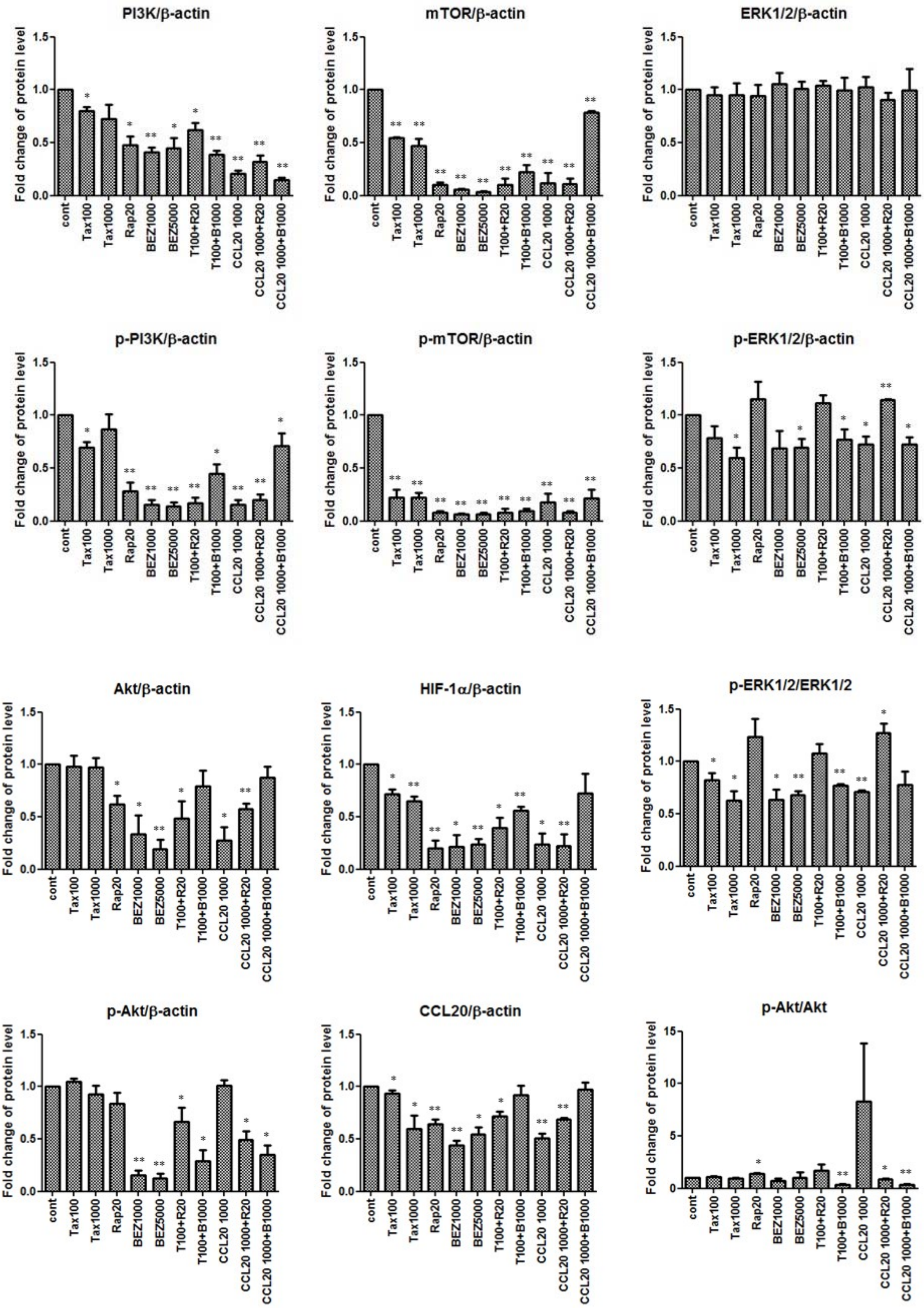
PI3K/β-actin
mTOR/β-actin
ERK1/2/β-actin
p-PI3K/β-actin
p-mTOR/β-actin
p-ERK1/2/β-actin
Akt/β-actin
HIF-1α/β-actin
p-ERK1/2/ERK1/2
p-Akt/β-actin
CCL20/β-actin
p-Akt/Akt
Fold change of protein level
cont
Tax100
Tax1000
Rap20
BEZ1000
BEZ5000
T100+R20
T100+B1000
CCL20 1000
CCL20 1000+R20
CCL20 1000+B1000

(D) SAS 48H

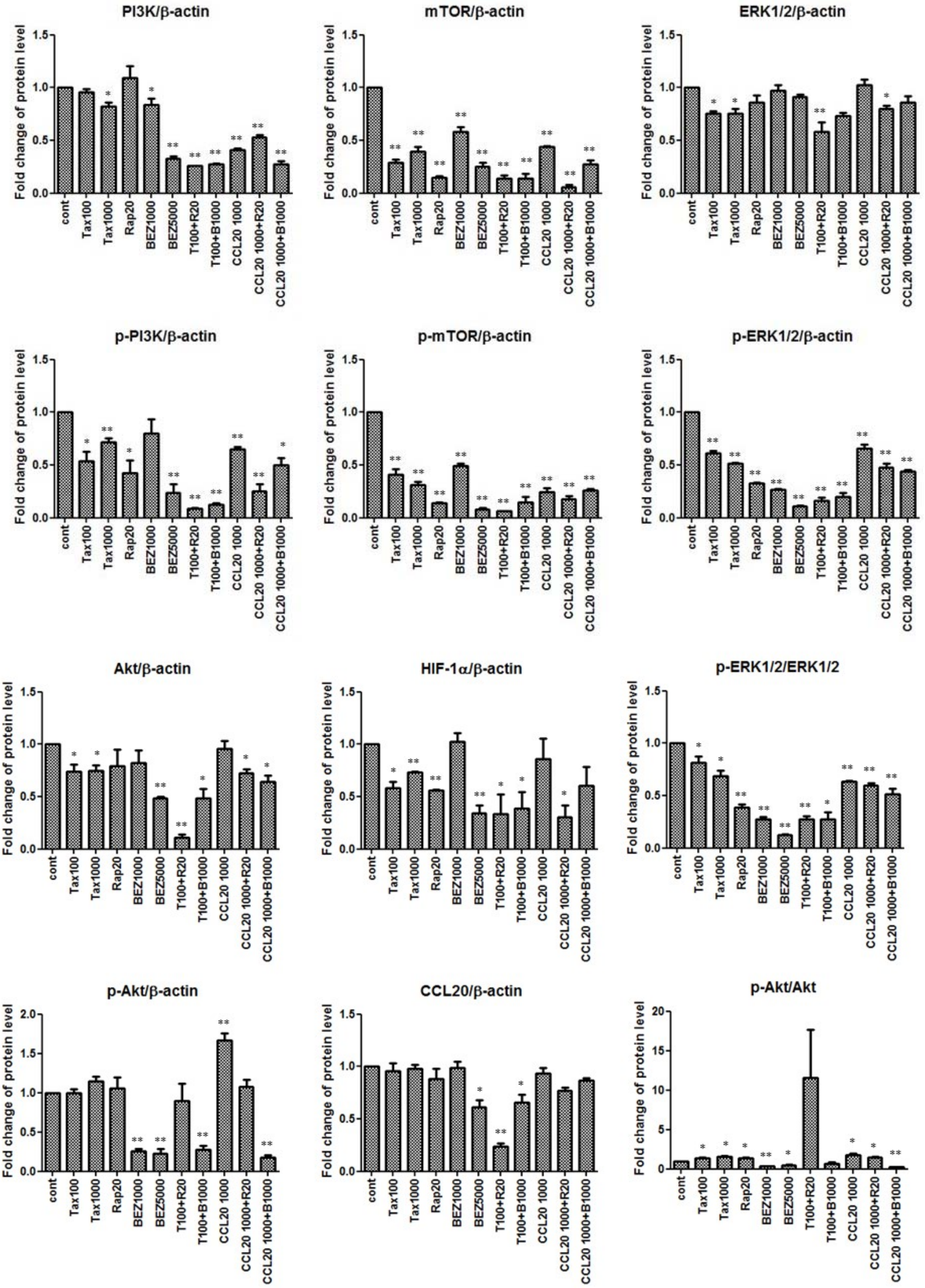
PI3K/β-actin
mTOR/β-actin
ERK1/2/β-actin
p-PI3K/β-actin
p-mTOR/β-actin
p-ERK1/2/β-actin
Akt/β-actin
HIF-1α/β-actin
p-ERK1/2/ERK1/2
p-Akt/β-actin
CCL20/β-actin
p-Akt/Akt
Fold change of protein level
cont
Tax100
Tax1000
Rap20
BEZ1000
BEZ5000
T100+R20
T100+B1000
CCL20 1000
CCL20 1000+R20
CCL20 1000+B1000

Figure S3. Effect of mTOR inhibitors (rapamycin and BEZ235) combined with docetaxel or CCL-20 on the expression of mTOR signaling pathway proteins in HNSCC cells. Cells were treated with docetaxel (0.1 or 1μM), rapamycin (20μM), BEZ-235 (1 or 5 μM), CCL-20 (1μg/mL) or the combination. After 24 hours or 48 hours, total cell lysates were prepared to perform Western blotting analysis for p-PI3K, PI3K, p-mTOR, mTOR, p-ERK1/2, ERK1/2, p-AKT S473, AKT, HIF-1α and CCL-20 expression. β-actin was used for loading control. (A, B) In the FaDu cell line, the marked reduction in mTOR signaling pathway protein levels after treatment with rapamycin or BEZ235 for 24 hours or 48 hours was synergistic with docetaxel and restored by CCL-20 treatment. Unlike BEZ235 (a dual inhibitor of PI3K and mTOR), rapamycin (20 μM) increased p-PI3K and p-ERK1/2 expressions at 24 hrs. (C, D) In the SAS cell line, the marked reduction in p-PI3K, p-ERK1/2, p-AKT S473 and mTOR signaling pathway protein levels after treatment with rapamycin or BEZ235 for 24 hours or 48 hours was synergistic with docetaxel and restored by CCL-20 treatment. Densitometric analysis results are means ± SEM for three independent experiments. $^{*}p<0.05$, $^{**}p<0.001$

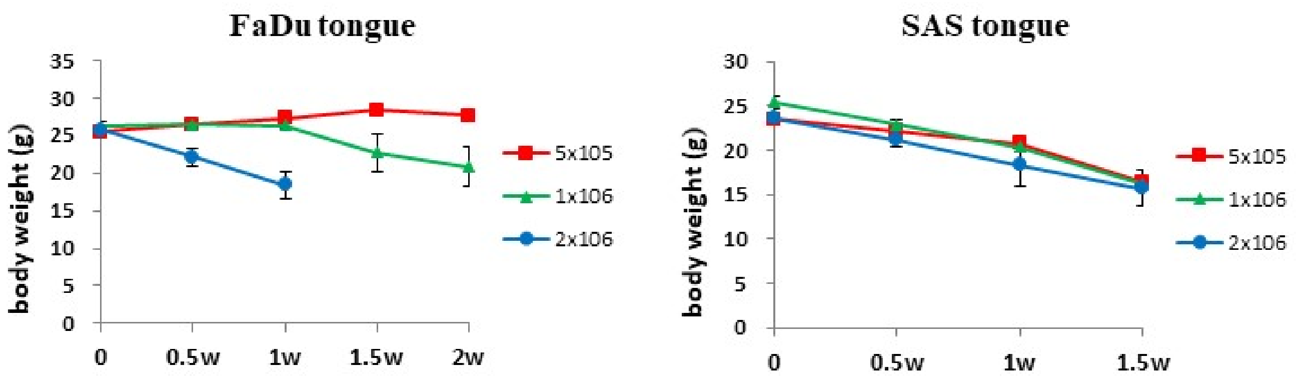

Figure S4. Body weight. Orthotopic xenograft mice were measured twice a week for 2 weeks and humanely euthanized by CO_2 inhalation when these mice had lost more than 25% of their pre-injection body weight. Data are mean values and error bars represent±standard error of the mean (SEM).
